# Effect of yellow sweetclover (*Melilotus officinalis*) hay compared with Lucerne (*Medicago sativa*) hay on carcass characteristics and meat quality of male goat kids

**DOI:** 10.5455/javar.2022.i631

**Published:** 2022-12-31

**Authors:** Lahkim Bennani Mouad, Aarab Ahmed, Jaber Abdelaziz, Acherkouk Mohamed, Ayadi Mohammed

**Affiliations:** 1 Research Team of Biotechnology and Biomolecular Engineering (ERBGB), Faculty of Sciences and Technology, Tangier (FSTT), Tangier, Morocco; 2 Faculty of Sciences and Technology, Research Team of Biotechnology and Biomolecular Engineering (ERBGB), Abdelmalek Essaadi University, Tangier, Morocco

**Keywords:** Carcass characteristics, goat-kid, growth performance, meat quality, Melilotus officinalis

## Abstract

**Objective:**

*Melilotus officinalis* is a plant that grows naturally in northwestern Morocco and could become a promising alternative. The study was carried out to investigate the effects of *M. officinalis* hay on growth performance, carcass characteristics, and meat quality of goat kids in northern Morocco compared to *Medicago sativa*.

**Materials and Methods:**

Eighteen 3-month-old male “Beni Arouss” goat kids have been divided similarly into two groups. The control group (Luc) received lucerne hay, and the test group (YSClov) received yellow sweetclover hay, both supplemented with concentrate. Average daily gain and dry matter intake were determined during the experiment. After 99 days, goat kids were weighed, and carcass characteristics were determined. Meat quality was evaluated using samples from the semimembranosus (SM) and longissimus thoracis muscles.

**Results:**

The addition of YSClov significantly increased ash content (*p* < 0.001) and fat content (*p* < 0.01), reduced water holding capacity (*p* < 0.01), and SM pH 24 (*p* < 0.05). The YSClov meat was significantly more tender than the Luc meat, with corresponding values of 8.20 and 11.80 kg/cm^2^ (*p* < 0.05), while the Luc meat was more tender when cooked. No significant effect was found for the other parameters. The YSClov meat is richer in desirable fatty acids (DFA), while the Luc meat appears to be richer in omega-3 DFA(*p* < 0.01).

**Conclusion:**

*Melilotus officinalis* hay showed promising results in intramuscular fat, protein content, tenderness, DFA content, and similar growth performance compared to conventional feeds.

## Introduction

The number of goats worldwide is about 1 billion animals [1]. In northwestern Morocco, the goat population approximates 788,000 animals, of which 70% are raised within the mountains of Chefchaouen and Tetouan provinces [2]. Due to the low feed availability in the region, livestock systems are characterized by low productivity and, therefore, low income compared to systems in the northern Mediterranean region [3,4].

Nowadays, consumers are paying more attention to their health, as the risk of contracting diseases is highly dependent on the quality of food [5]. Goat meat has a better nutritional quality compared to other red meats, with more tender meat and a higher content of polyunsaturated fatty acids (PUFA) [5,6].

Yellow sweetclover (*Melilotus officinalis* (L.) Lam.) is an annual or biennial herb native to Eurasia [7]. Several studies have shown the antioxidant activity of *M. officinalis* [8] since it may contain bioactive compounds such as coumarin and flavonoids [9,10].

Diet can influence the FA composition of ruminants [11]. Feeds rich in phenolic compounds, unlike cereals, inhibit fatty acids’ (FA) biohydrogenation in the rumen and subsequently promote better incorporation of unsaturated FAs into muscle tissue [12]. Moreover, adding flavonoids minimizes lipid oxidation in sheep meat [13].

No studies have been conducted on introducing *M. officinalis* hay into goat diets. In this context, the objective is to assess the effects of incorporating *M. officinalis* hay admixture in the diet of kid goats on growth performance, carcass characteristics, and meat quality.

## Materials and Methods

### Ethical approval

Ethical housing, feeding, and handling were approved by the National Institute of Agronomic Research-Regional Center of Tangier (permit number: 935/INRA/DGRHF/DC/3). All efforts had been made to reduce the animals’ suffering.

### Animals and feeding management

This study was carried out at the experimental farm of the National Institute of Agronomic Research in Tangier (INRA-Morocco) (35°39’N, 5°51’W; 11 m south latitude). Eighteen 3-month-old male goat kids of the indigenous “Beni Arouss” breed with a preliminary weight of 11.05 ± 0.97 kg were similarly divided into two groups based on age and body weight. The experimental period was 99 days, which included a 9-day adaptation period. The control group (Luc) received lucerne hay as roughage, which was replaced by yellow sweetclover hay in the experimental group (YSClov). *Melilotus officinalis* was grown at the INRA experimental station, air-dried for 10 days, and packed. Both groups received the same specific amount of concentrated supplements. Table 1 shows the ingredients and proportions in the concentrate, the nutrient composition in 1 kg dry matter (DM), and the FA profile of each feed. The amount administered was adjusted considering the weight evolution of the kid goats according to the French feeding system [14], and the food refusal was not significant. The average daily gain(ADG) was calculated during the experiment. The fed and refused feed was weighed daily for each group to determine DM intake (DMI). Yellow sweetclover and lucerne hay were ground and mixed with concentrate twice daily before feeding. The food consumption ratio (FCR) was measured using the following formula: FCR = DMI/ADG.

### Carcass characteristics

The kid goats were instantly weighed at the end of the experiment and then slaughtered at the INRA abattoir, following the recommended hygiene procedures [15]. Immediately after slaughter, post-mortem measurements were performed, including hot carcass weight and empty carcass weight, carcass length (CL) for the calculation of compactness index (CI = CW/CL), and thigh length (TL) and thigh thickness (TT) for the calculation of muscle index (MI = TT/TL) and conformation index (CfI = CI + MI).

Carcasses were stored in a cold room at 4°C for 24 h. Then, the cold carcasses were weighed, and samples of the longissimus thoracis (LT) and semimembranosus (SM) muscles were taken from each carcass to evaluate meat quality.

### Meat quality

At 24 h post-mortem, sampling was done from LT and SM. The coloration of LT was obtained immediately after sampling using the Konica-Minolta CR400® colorimeter. The color was measured according to the CIE standard [16]. Samples were then ground to measure moisture content and water holding capacity (WHC%) for both samples and to determine ash, protein, and fat content. Samples were labeled, packaged, and stored at −25°C for chemical analysis.

**Table 1.** Ingredients, nutritive composition, and FAs profile of goats’ diets.

	YSClov	Luc
Ingredients (%)		
Yellow sweetclover hay	52	0
Lucerne hay	0	56
Orge (%)	14	13
Corn (%)	17	16
Wheat bran (%)	5	4
Soybean (%)	12	11
FU_meat_ (gm/kg DM)	1.10	1.14
Digestible proteins in the intestines (gm/kg DM)	138	130
Ca (gm/kg DM)	9.67	6.57
P (gm/kg DM)	3.87	4.67
Chemical composition (gm/kg DM)		
Dry matte (DM)	900	900
Ash	139	142
Crude protein	205	200
Ether extract	46.4	40.0
Crude fiber (gm)	333	215
Ca (gm/kg DM)	9.67	6.57
P (gm/kg DM)	3.87	4.67
Forage unit for meat (FU meat gm/kg DM)	1.10	1.14
Digestible proteins in the intestines (gm/kg DM)	138	119
FAs (gm/100 gm FAs)		
Palimitic C16:0	6.00	0.00
Trans-Palmitelaidic C16:1	0.40	0.00
Stearic C18:0	1.51	0.11
Oleic C18:1n9c	22.0	4.13
Eicosenic C20:1	0.55	0.00
Trans-Linoleic C18:2n6t	17.1	15.0
Cis-Linoleic C18:2n6c	24.1	2.99
γ –Linolenic C18:3n6	17.9	25.4
α-Linolenic C18:3n3	0.69	0.24
Arachidic C20:0	0.13	0.00
Eicosadienoic C20:2	1.28	14.0
Eicosatrienoic C20:3n3	0.18	0.19
Erucic C22:1n9	0.00	6.27

YSClov: Yellow sweetclover Group; Luc: Lucerne Group.

Prior to sampling, pH 0 and pH 24 of the meat were obtained using HANNA HI99163 pH meter for both muscles. Moisture content was calculated for both samples [17]. Ash was obtained at 550°C for 5 h. WHC% was determined by pressing the sample weighing 2.250 kg between two filter papers for 5 min [18].

The fat content was determined using the Soxhlet method (AOAC, 1997, ID 920.39). Protein content was determined following the Kjeldahl method (AOAC, 1997, ID 955.04). Shear force was determined using the Warner-Bratzler shear force protocol [19] on LT samples. The meat samples used for shear force measurement had a size of 10*10*30 mm^3^ and a weight of about 10 gm, were cylindrical, and the shear cut was performed three times perpendicular to the grain direction [5]. Shear force was determined for raw and cooked meat. Cooked meat was heated in a glass tube (30*100 mm) and placed in a water bath at 75°C for 40 min.

### FAs profile

The extraction of intramuscular fat was performed using the chloroform:methanol (2 v:v) method described previously [20]. FAs were identified using a standard (FAME Sigma-Aldrich), which refers to 37 FAs. The groups, ratios, and indexes were calculated using the formulas reported by Banskalieva et al. [5].

### Statistical analysis

Data were processed using SAS^®^ version 9.1 software [21]. The effect of the dietary factor was tested with a one-way analysis of variance.

## Results and Discussion

Table 2 shows the diet’s effect on the production and carcass characteristics of the kid goats. After 3 months of the experiment, the diet did not affect those parameters, and the kids had the same weight.

Observations on carcass characteristics are presented in Table 3. Diet affects the MI (*p* < 0.05), with 0.22 for Luc and 0.20 for YSClov. No unusual values were found for the other parameters.

The results in Table 4 indicate the diet’s effect on the nutritional and technological parameters of the meat of kid goats for the Luc and YSClov groups. It was found that the raw meat of YSClov appeared to be more tender than that of Luc (8.20 *vs.* 11.80 kg/cm^2^) and richer in ash and fat (17.51 *vs.* 15.86 gm/kg and 38.01 *vs.* 25.90 gm/kg, respectively) (*p* < 0.01). YSClov showed a lower WHC content of 24.16% and 22.22% for the samples SM and LT, respectively (*p* < 0.05). In terms of color, a* and b* were found to increase and L* to decrease over the 12–24-h period.

**Table 2.** The effect of diet on growth performances of goat kids (*n* = 9).

	Luc	YSClov	SEM	*p*-value
DM ingested (gm/head/day)	1,305	1,253	141	0.796
Initial body weight (kg)	12.8	12.1	1.52	0.767
Final body weight (kg)	19.8	19.5	1.95	0.908
ADG_90–180_ (gm/day)	78.4	81.9	10.49	0.809
FCR	10.8	9.91	1.07	0.728

Luc: Lucerne Group; YSClov: Yellow sweetclover Group; ADG: Average Daily Gain*; FCR *(*Feed consumption ratio*): DMI/ADG dry matter intake/average dairy gain; SEM: Standard mean error.

**Table 3.** Effect of diet on carcass characteristics of goat kids (*n* = 9).

	Luc	YSClov	SEM	*p*-value
Cold carcass weight (kg)	9.66	9.87	1.14	0.897
Carcass yield (%)	52.9	55.7	7.92	0.807
Perirenal fat (gm)	159	152	27.44	0.856
Mesenteric fat (gm)	584	593	109.65	0.953
CL (cm)	49.4	49.7	1.87	0.931
TL (cm)	36.2	36.5	1.67	0.879
TT (cm)	7.96	7.39	0.26	0.142
CI	0.19	0.19	0.02	0.962
MI	0.22	0.20	0.01	0.270
Conformation score	0.41	0.40	0.02	0.589
Cover fat color score	5.38	5.42	0.28	0.909

Luc: Lucerne Group; YSClov: Yellow sweetclover Group; SEM Standard mean error.

As shown in Table 5, kids from both groups were rich in oleic acid cis (C18:1n9c) and linoleic acid cis (C18:2n6c). Diet significantly affected the content of some FAs (*p* < 0.05). The Luc was richer in C20:2, c24:0, C18:3n3, c20:3n3, C20:5n3, and C21:0, while the YSClov was richer in C16:0 and C18:0 (*p* < 0.05). As shown in Table 6, the effect of diet on some FA groups was significant (*p* < 0.05). The YSClov group appeared to be richer in desirable FA (DFA), while the Luc group was richer in omega-3 FAs.

In general, the performance of the goat kids was the same in both groups, indicating that diet had no significant influence. The FBW is considered an important indicator of CCW and CY% [22].

These values are higher compared to those reported by El Otmani et al. [23] for the “Beni Arouss” breed and are close to the results published by Ayadi et al. [24] for the same breed. In general, low protein content has a limiting effect on growth performance [25].

The carcass yield obtained in this study was higher than the results reported by Xie et al. [26] for male Kashmir goats of the Jin-lan breed and those reported by Kafle et al. [27] for male goats of the Kiko breed. Typically, the carcass yield of goats ranges from 49% to 51% [28]. This high value can be explained by the halal method followed by the Muslim rituals for slaughter, which was applied in our experiment, allowing a better emptying of the blood [28]. The results of the conformity index are in agreement with those found by El Otmani et al. [23] for the Beni Arouss breed, with a higher CI (0.11) and a lower MI (0.29).

**Table 4.** Diet’s effect on dietary and technological parameters of goat kids’ meat (*n* = 9).

Parameters	Luc	YSClov	SEM	*p*-value
Ash (gm/kg)	15.9	17.5	l0.26	**<0.001**
SM moisture (%)	79.5	79.1	0.28	0.364
LT moisture (%)	78.8	79.1	0.32	0.443
Fat (gm/kg)	25.9	38.0	2.85	**0.006**
CP (gm/kg)	206	209.6	2.79	0.359
SM pH_0_	6.21	6.22	0.04	0.865
SM pH_24_	5.91	5.81	0.03	**0.048**
LT pH_0_	6.69	6.46	0.04	**<0.001**
LT pH_24_	5.96	5.91	0.05	0.401
SM WHC (%)	31.7	24.2	1.57	**0.002**
LT WHC (%)	25.7	22.2	1.07	**0.038**
L* *LT *12 h	46.1	47.2	1.10	0.478
a* *LT *12 h	18.0	17.8	0.54	0.829
b* *LT* 12 h	3.96	2.18	0.22	0.061
L* *LT *24 h	44.2	44.7	0.65	0.569
a* *LT *24 h	20.7	19.8	0.51	0.236
b* *LT* 24 h	3.96	3.21	0.30	0.105
Shear force raw meat (kgf/cm^2^)	11.8	8.20	0.55	**<0.001**
Shear force cooked meat (kgf/cm²)	4.85	5.81	0.25	**0.011**

YSClov: Yellow sweetclover Group, Luc: Lucerne Group, LT: Longissimus thoracis*, *SM: *Semimembranosus, *L*: lightness index; a*: redness index; b*: yellowness index, WHC: Water holding capacity, SEM: Standard mean error.

*p*-value < 0.05 shows a significant effect, and bold values show a significant effect.

The values obtained for perirenal and mesenteric fat showed no significant influence from the diet. Moreover, these values were higher than those obtained for the same breed with a less energy-rich diet [23]. Indeed, the energetic compounds’ content profoundly affects the development of adipose tissue [29].

Diet does not affect color values and is close to the values obtained for goat meat in other studies [30,31]. Goat meat was darker in color than other red meat [30]. Both groups showed lower brightness and higher redness compared to Spanish goats [32]. These results could be due to more type I muscle fibers and a thicker perimysium in the “Beni Arouss” breed [33]. In general, the diet has no significant effect on coloration. Bjelanovic et al. [34] pointed out that the presence of vitamin E in the muscle indicates a stable pattern in the coloration of the meat, which could be the cause of the similarity of coloration between the two groups since this compound is present in the concentrate.

**Table 5.** Effect of diet on LTFAs profile of goat-kids (gm/100 gm FAs) (*n* = 9).

FAs	Luc	YSClov	SEM	*p*-value
C12 :0	0.49	0.17	0.04	0.613
C13 :0	0.11	0.17	0.03	0.188
C14 :0	0.20	0.16	0.03	0.347
C16 :0	18.7	22.3	1.31	**0.014**
C16 :1	0.87	0.79	0.15	0.713
C17 :1	0.54	1.04	0.08	0.611
C18 :0	12.2	15.7	1.33	**0.032**
C18 :1n9t	2.23	2.13	0.34	0.833
C18 :1n9c	25.3	26.5	1.48	0.620
C18 :2n6t	1.70	1.38	0.26	0.582
C18 :2n6c	7.43	9.74	1.19	0.131
C20 :0	0.48	0.26	0.08	0.091
C18 :3n6	2.40	1.96	0.74	0.682
C20 :1	1.41	1.45	0.57	0.963
C18 :3n3	0.67	0.16	0.09	**0.002**
C21 :0	1.17	0.29	0.20	**0.018**
C20 :2	0.71	0.43	0.08	**0.031**
C22 :0	0.29	0.22	0.04	0.177
C20 :3n6	0.64	0.27	0.14	0.105
C22 :1n9	0.39	0.35	0.06	0.649
C20 :3n3	0.30	0.088	0.10	**<0.001**
C23 :0	5.12	3.42	1.60	0.198
C20 :4n6	0.96	0.49	0.23	0.095
C22 :2	0.47	0.24	0.08	0.079
C24 :0	0.77	0.32	0.13	**0.030**
C20 :5n3	2.36	1.27	0.14	**<0.001**
C24 :1	1.42	1.29	0.30	0.793
C22 :6n3	1.79	1.71	0.19	0.807

Luc: Lucerne Group, YSClov: Yellow sweetclover Group, SEM: Standard mean error.

*p*-value < 0.05 shows a significant effect, and bold values show a significant effect.

The pH of the meat affects the meat quality [35]. Hamdi et al. [36] reported that pH 0 must be below 6.4 and pH 24 must be between 5.4 and 5.7 for the meat to be marketable. Only the pH0 of LT does not meet these criteria. Kids generally have a pH of 24, which is between 5.8 and 6.2. This is due to the excitable nature of the goat breed and the stress before death, which reduces glycogen reserves [6].

**Table 6.** Effect of diet on groups, ratios, and indexes of LT FAs profile of goat-kids (gm/100 gm fat) (*n* = 9).

FAs groups	Luc	YSClov	SEM	*p*-value
SFA	48.1	48.02	1.62	0.967
MUFA	30.2	31.3	1.75	0.677
PUFA	19.4	18.5	1.65	0.703
DFA	61.8	65.6	1.31	**0.025**
Omega-3	5.11	4.01	0.41	**0.043**
Omega-6	13.1	13.9	1.52	0.742
Omega-9	27.9	29.0	1.86	0.674
Ratios				
PUFA/SFA	0.40	0.42	0.04	0.807
MUFA/PUFA	1.82	2.06	0.25	0.483
Omega-6/omega-3	2.88	3.59	0.35	0.244
Indexes				
AI	0.41	0.49	0.04	0.089
(C18:0+C18:1)/C16:0	2.21	2.02	0.12	0.321

Luc: Lucerne Group, YSClov: Yellow sweetclover Group, SFA: Saturated fatty acids, PUFA: Poly-unsaturated fatty acids, MUFA: Mono-unsaturated fatty acids, DFA: Desirable fatty acids, AI: Atherogenicity Index; SEM: Standard mean error.

*p*-value < 0.05 shows a significant effect, and bold values show a significant effect.

Ash content is highly dependent on diet. Indeed, ash content may vary according to age, sex, weight, and diet [6]. Although ash content is higher in YSClov, these results are lower than those reported for the same breed [23,24,37] and range from 1.1% to 5.0% [38].

Diet exclusively affects WHC in LT and SM (*p* < 0.01). This could be due to post-mortem pH and intramuscular fat. Several authors reported that the variation in WHC was probably caused by post-mortem pH, carcass fat, meat protein, and intramuscular fat content [38,39]. No significant effects were observed for ADG, FBW, and CP; only post-mortem pH and intramuscular fat were significant between the two groups.

The type of FAs, juiciness, tenderness, and meat flavor make intramuscular fat more beneficial [40]. Fat content was strictly dependent on feeding. Nevertheless, alfalfa hay had a lower EE content, which may be attributed to the fact that YSClov received a relatively higher energy diet than Luc. Ivanovic et al. [41] and Karaca et al. [42] mentioned that a higher-energy diet positively affects the fat content of the meat.

Meat texture depends on several factors, especially pre-mortem temperature, post-mortem carcass texture, post-mortem pH, feed contribution, sample preparation, and glucose concentration in muscle [6,43]. It also depends on collagen content and muscle fiber size [44]. In fact, nutrition had a very significant effect on tenderness, as the results showed that it was positively correlated with LT pH (*r*^2^ = 0.5281). YSClov meat is more tender and remains within the norm of 8.3 and 8.4 kg/cm^2^ for goats [45]. Shear force was determined for the cooked meat, and it was found that the meat of Luc LT was more tender than that of YSClov. Nevertheless, both obtained excellent results and remained within the norm mentioned below. These results could be due to the fat content loss after cooking. Fat deposition affects glycogen availability at slaughter [46], and intramuscular fat may affect tenderness [47], which is consistent with the present results.

The profile of ingested nutrients may affect protein content and fat independently of ADG [48]. Nevertheless, meat protein is rarely found to change [49,50]. Migdał et al. [51] and Silva et al. [52] reported the same value.

The FA profile impacts meat’s high satisfaction and, for this reason, has an effective impact on human health [6,53]. It is widely known that goat meat contains palmitic acid (C16:0), stearic acid (C18:0), oleic acid (C18:1), and linoleic acid (C18:2), which makes it very healthy.

This study showed that the FAs cited above are within the range reported by Banskalieva et al. [5]. The intramuscular fat is rich in oleic acid cis C18:1n9c and linoleic acid cis C18:2n6c; these values are due to the high content of these compounds in the diets of both groups. The association of the Δ5- and Δ6-desaturase enzymes with elongase can synthesize long-chain PUFA from C18:3n3 and C18:3n6. This could explain their higher content in the Luc group [54].

Generally, beef and sheep meat had a higher DFA content than goat meat. In this study, the DFA content agreed with the margin of (61%–80%) reported by Banskalieva et al. [5].

The results for saturated fatty acids (SFA) content were largely above those reported by Ayadi et al. [24] and below those reported by El Otmani et al. [23] for the same breed. SFAs were slightly lower in the YSClov group. Ayadi et al. [24] used condensed doses of tannins that deactivate biohydrogenation [53]. Yellow sweetclover contains coumarin [55] and flavonoids. Purba et al. [56] and Resconi et al. [57] reported that flavonoid-rich forages decreased SFA concentrations and improved UFA. Lucerne contains more protein and xanthophyll [58], which explains its high omega-3 FA content. Dang Van et al. [59] reported that PX increased omega-3 FAs in cow milk.

Both groups showed excellent results for the ratio of PUFA to SFA. Wood et al. [60] recommend a value of 0.4 for human health; a lower value causes cholesterol. Sweetclover contains flavonoids [61], and a study has shown that this component can alter the cholesterol content and FA profile in ruminant meat [62,63]. Antioxidant-rich feeds increase PUFA content [60].

The enzyme Δ-6-desaturase converts competitor omega-3 and omega-6 to long-chain PUFA. However, the Δ-6-desaturase prefers omega-3 [64], which is in contradiction with the obtained results. This may be due to the high content of C18:3n6 in the concentrate. Indeed, a concentrate rich in n-6 linoleic acid increases the ratio of omega-6 to omega-3 [65]. Demirel et al. [66] reported that a diet high in PUFA/SFA and omega-6/omega-3 ratios affects these compounds in intramuscular fat by 25%-75%.

## Conclusion

In this study, the effects of adding *M. officinalis* to the diet of kid goats on growth performance, carcass characteristics, and meat quality are investigated. In general, it has no effect on carcass performance parameters or some meat quality parameters. However, *M. officinalis* showed good results in important parameters for the evaluation of meat by consumers, such as tenderness, protein content, and intramuscular fat. Furthermore, incorporating sweet clover into the diet increases the DFAs. These results could be promising for the northwestern region of Morocco, and yellow sweetclover could be considered as a potential alternative for breeders to ensure a balanced diet throughout the year and also to minimize fattening costs.

## List of abbreviations

Luc, Control group received lucerne hay; YSClov, Test group received yellow sweetclover hay; ADG, Average daily gain; DMI, Dry matter intake; SM, semimembranosus; LT, longissimus thoracis; WHC, Water holding capacity; DFA, Desirable fatty acids; MUFA, Monounsaturated fatty acids; PUFA, Polyunsaturated fatty acids; FCR, Food consumption ratio; CI, Compactness Index; TL, Thigh length; TT, Thigh thickness; MI, Muscle Index; CY%, Carcass yield; CfI, Conformation Index; L*, lightness index; a*, redness index; b*, yellowness index; FBW, Final body weight; CP, Crude protein.

## References

[1] FAO (2017). FAOSTAT, Food and Agriculture Organization of the United Nations. http://www.fao.org/faostat/en/#data/QA.

[2] Chentouf M, Bister JL, Boulanouar B (2011). Reproduction characteristics of North Moroccan indigenous goats. Small Rumin Res.

[3] Tegegne F, Mondragón-Jacobo C, Pérez-González S (2001). Nutritional value of *Opuntia ficus-indica* as a ruminant feed in Ethiopia. Cactus (*Opuntia* spp.).

[4] Chentouf M, Zantar S, Doukkali M, Farahat L, Jouamaa A, Aden H, Bernués A, Boutonnet JP, Casasús I, Chentouf M, Gabiña D, Joy M (2011). Performances techniques et économiques des élevages caprins dans le nord du Maroc (Options Méditerranéennes Série A. Séminaires Méditerranéens). Economic, social and environmental sustainability in sheep and goat production systems.

[5] Banskalieva V, Sahlu T, Goetsch AL (2000). Fatty acid composition of goat muscles and fat depots: a review. Small Rumin Res.

[6] Webb EC, Casey NH, Simela L (2005). Goat meat quality. Small Rumin Res.

[7] Wu SH, Chaw SM, Rejmánek M (2003). Naturalized *Fabaceae* (Leguminosae) species in Taiwan: the first approximation. Bot Bull Acad Sin.

[8] Trouillas P, Calliste CA, Allais DP, Simon A, Marfak A, Delage C (2003). Antioxidant, anti-inflammatory and antiproliferative properties of sixteen water plant extracts used in the Limousin countryside as herbal teas. Food Chem.

[9] Al-Snafi AE (2020). Chemical constituents and pharmacological effects of *Melilotus officinalis*—a review. Curr Pharmacol Rep.

[10] Ilhan M, Ali Z, Khan IA, Taştan H, Küpeli Akkol E (2020). The regression of endometriosis with glycosylated flavonoids isolated from *Melilotus officinalis* (L.) Pall. in an endometriosis rat model. Taiwan J Obstet Gynecol.

[11] Vargas-Bello-Pérez E, Larraín R E (2017). Impacts of fat fromruminants’ meat on cardiovascular health and possible strategies to alter its lipid composition. J Sci Food Agric.

[12] Salami SA, Luciano G, O’Grady MN, Biondi L, Newbold CJ, Kerry JP (2019). Sustainability of feeding plant by-products: a review of the implications for ruminant meat production. Anim Feed Sci Technol.

[13] Simitzis PE, Charismiadou MA, Goliomytis M, Charalambous A, Ntetska I, Giamouri E (2019). Antioxidant status, meat oxidative stability and quality characteristics of lambs fed with hesperidin, naringin or α-tocopheryl acetate supplemented diets. J Sci Food Agric.

[14] INRA (2018). INRA feeding system for ruminants. INRA.

[15] Duan T, Wu Z, Zhang H, Liu Y, Li Y, Zhang W (2019). Effects of melatonin implantation on carcass characteristics, meat quality and tissue levels of melatonin and prolactin in Inner Mongolian cashmere goats. J Anim Sci Biotechnol.

[16] Commission Internationale de l’Eclairage (2004). Ocular lighting effects on human physiology and behaviour.

[17] AOAC (1997). Official methods of analysis of AOAC International.

[18] Farouk MM, Wieliczko KJ (2003). Effect of diet and fat content on the functional properties of thawed beef. Meat Sci.

[19] Association American Meat Science (1995). Research guidelines for cookery, sensory evaluation, and instrumental tenderness measurements of meat.

[20] Chaosap C, Sivapirunthep P, Takeungwongtrakul S, Zulkifli RM, Sazili AQ (2020). Effects of Zn-L-selenomethionine on carcass composition, meat characteristics, fatty acid composition, glutathione peroxidase activity, and ribonucleotide content in broiler chickens. Food Sci Anim Resour.

[21] SAS Institute (2004). SAS version 9.1.

[22] Bauman DE, Perfield JW, De Veth MJ, Lock AL (2003). New perspectives on lipid digestion and metabolism in ruminants.

[23] El Otmani S, Chebli Y, Hornick JL, Cabaraux JF, Chentouf M (2020). Growth performance, carcass characteristics and meat quality of male goat kids supplemented by alternative feed resources: olive cake and cactus cladodes. Anim Feed Sci Technol.

[24] Ayadi M, Arakrak A, Chriyaa A, El Otmani S, Chentouf M, Zantar S (2014). Effets des tanins condensés de la pulpe de caroube sur la production et la qualité du lait et de la viande caprine. Options Méditerr Ser A Semin Mediterr.

[25] Zhu W, Xu W, Wei C, Zhang Z, Jiang C, Chen X (2020). Effects of decreasing dietary crude protein level on growth performance, nutrient digestion, serum metabolites, and nitrogen utilization in growing goat kids (*Capra hircus*). Animal (Basel).

[26] Xie B, Wang PJ, Yan ZW, Ren YS, Dong KH, Song ZP (2018). Growth performance, nutrient digestibility, carcass traits, body composition, and meat quality of goat fed Chinese jujube (*Ziziphus Jujuba Mill*) fruit as a replacement for maize in diet. Anim Feed Sci Technol.

[27] Kafle D, Lee JH, Min BR, Kouakou B (2021). Carcass and meat quality of goats supplemented with tannin-rich peanut skin. J Agric Food Res.

[28] Turner KE, Cassida KA, Zerby HN (2014). Meat goat kids finished on alfalfa, red clover or orchardgrass pastures: carcass merit and meat quality. Meat Sci.

[29] Corazzin M, Del Bianco S, Bovolenta S, Piasentier E, Lorenzo JM, Munekata PES, Barba FJ, Toldrá F (2019). Carcass characteristics and meat quality of sheep and goat. More than beef, pork and chicken—the production, processing, and quality traits of other sources of meat for human diet.

[30] Pophiwa P, Webb EC, Frylinck L (2017). Carcass and meat quality of Boer and indigenous goats of South Africa under delayed chilling conditions. South Afr J Anim Sci.

[31] Guzmán JL, De La Vega F, Zarazaga LÁ, Argüello A, Delgado-Pertíñez M (2019). Carcass characteristics and meat quality of conventionally and organically reared suckling dairy goat kids of the Payoya breed. Ann Anim Sci.

[32] Batchuv P, Hazard T, Lee JH, Terrill TH, Kouakou B, Kannan G (2021). High-condensed tannin diet and transportation stress in goats: effects on physiological responses, gut microbial counts and meat quality. Animals.

[33] Bakhsh A, Hwang YH, Joo ST (2019). Effect of slaughter age on muscle fiber composition, intramuscular connective tissue and tenderness of goat meat during post-mortem time. Foods.

[34] Bjelanovic M, Grabez V, Vucic G, Martinovic A, Lima LR, Markovic B (2015). Effects of different production systems on carcass and meat quality of sheep and lamb from Western Balkan and Norway. Biotechnol Anim Husb Biotehnol Stoc.

[35] Guerrero A, Campo M, Olleta JL, Sañudo C, Kukovics S (2019). Carcass and meat quality in goat. Goat science, Intech Open.

[36] Hamdi H, Majdoub-Mathlouthi L, Picard B, Listrat A, Durand D, Znaïdi IA (2016). Carcass traits, contractile muscle properties and meat quality of grazing and feedlot Barbarine lamb receiving or not olive cake. Small Rumin Res.

[37] Ayadi M, Arakrak A, El Otmani S, Chentouf M (2016). Effet de différentes doses de Polyéthylène glycol sur la production et la qualité de la viande de chevreaux recevant un concentré riche en tanins condensés. Options Mediterr Ser A Semin Mediterr.

[38] Brand TS, van der Westhuizen EJ, van Der Merwe DA, Hoffman LC (2018). Analysis of carcass characteristics and fat deposition of Merino, South African Mutton Merino and Dorper lambs housed in a feedlot. South Afr J Anim Sci.

[39] Freitas HS, Alcalde CR, Lima LS, Macedo F, Macedo VD, Molina BS (2011). Quantitative characteristics of carcass and meat quality of ¾ Boer + ¼ Saanen and Saanen goat kids fed diets with dry yeast. Rev Bras Zootec.

[40] Frank D, Joo ST, Warner R (2016). Consumer acceptability of intramuscular fat. Korean J Food Sci Anim Resour.

[41] Ivanovic S, Pavlovic I, Pisinov B (2016). The quality of goat meat and it’s impact on human health. Biotechnol Anim Husb.

[42] Karaca S, Yilmaz A, Kor A, Bingöl M, Cavidoğlu I (2011). nd it’s impact on human health. Rev Bras Zootec.

[43] Cordova-Torres AV, Costa RG, Araújo Filho JT, Medeiros AN, Andrade-Montemayor H M (2017). Meat and milk quality of sheep and goat fed with cactus pear. J Prof Assoc Cactus Dev.

[44] Berthelot V (2018). Alimentation des animaux et qualité de leurs produits. Lavoisier, Paris, France,.

[45] Santos VAC, Silva SR, Azevedo JMT (2008). Carcass composition and meat quality of equally mature kids and lambs. J Anim Sci.

[46] Abdullah AY (2007). Musallam HS. Effect of different levels of energy on carcass composition and meat quality of male black goats kids. Livest Sci.

[47] Joven M, Pintos E, Latorre MA, Suárez-Belloch J, Guada JA, Fondevila M (2014). Effect of replacing barley by increasing levels of olive cake in the diet of finishing pigs: Growth performances, digestibility, carcass, meat and fat quality. Anim Feed Sci Technol.

[48] Wuliji T, Goetsch AL, Sahlu T, Puchala R, Soto-Navarro S, Merkel RC (2003). Effects of different quality diets consumed continuously or after a lower quality diet on characteristics of growth of young Spanish goats. Small Rumin Res.

[49] de Araújo TLAC, Pereira ES, Mizubuti IY, Campos ACN, Pereira MWF, Heinzen EL (2017). Effects of quantitative feed restriction and sex on carcass traits, meat quality and meat lipid profile of Morada Nova lambs. J Anim Sci Biotechnol.

[50] Ribeiro RDX, Medeiros AN, Oliveira RL, de Araújo GGL, Queiroga R, Ribeiro MD (2018). Palm kernel cake from the biodiesel industry in goat kid diets. Part 2: Physicochemical composition, fatty acid profile and sensory attributes of meat. Small Rumin Res.

[51] Migdał W, Kawęcka A, Sikora J, Migdał Ł (2021). Meat quality of the native carpathian goat breed in comparison with the Saanen breed. Animals.

[52] Silva WP, Santos SA, Cirne LGA, Pina D, Alba HDR, Rodrigues TCGC (2021). Carcass characteristics and meat quality of feedlot goat kids fed high-concentrate diets with licury cake. Livest Sci.

[53] Kotsampasi, Bampidis VA, Tsiaousi A, Christodoulou C, Petrotos K, Amvrosiadis I (2017). Effects of dietary partly destoned exhausted olive cake supplementation on performance, carcass characteristics and meat quality of growing lambs. Small Rumin Res.

[54] Wood JD, Enser M, Fisher AV, Nute GR, Sheard PR, Richardson RI (2008). Fat deposition, fatty acid composition and meat quality: a review. Meat Sci.

[55] Hooser SB, Wilson CR (2018). Comparative hepatotoxicology. Compr Toxicol.

[56] Purba RAP, Yuangklang C, Paengkoum S, Paengkoum P (2020). Milk fatty acid composition, rumen microbial population and animal performance in response to diets rich in linoleic acid supplemented with Piper betle leaves in Saanen goats. Anim Prod Sci.

[57] Resconi VC, Pascual-Alonso M, Aguayo-Ulloa L, Miranda-De La Lama GC, Alierta S, Campo MM (2018). Effect of dietary grape pomace and seed on ewe milk and meat quality of their suckling lambs. J Food Qual.

[58] Pietrzak KGE (2014). Production technology, chemical composition and use of Alfalfa protein-xanthophyll concentrate as dietary supplement. J Food Process Technol.

[59] Dang Van QC, Bejarano L, Mignolet E, Coulmier D, Froidmont E, Larondelle Y (2011). Effectiveness of extruded rapeseed associated with an alfalfa protein concentrate in enhancing the bovine milk fatty acid composition. J Dairy Sci.

[60] Wood JD, Richardson RI, Nute GR, Fisher AV, Campo MM, Kasapidou E (2004). Effects of fatty acids on meat quality: a review. Meat Sci.

[61] Wang S, Yu Z, Wang C, Wu C, Guo P, Wei J (2018). Chemical constituents and pharmacological activity of agarwood and aquilaria plants. Molecules.

[62] Andrés S, Morán L, Aldai N, Tejido ML, Prieto N, Bodas R (2014). Effects of linseed and quercetin added to the diet of fattening lambs on the fatty acid profile and lipid antioxidant status of meat samples. Meat Sci.

[63] Tan CY, Zhong RZ, Tan ZL, Han XF, Tang SX, Xiao WJ (2011). Dietary inclusion of tea catechins changes fatty acid composition of muscle in goats. Lipids.

[64] Simopoulos AP (2010). Genetic variants in the metabolism of omega-6 and omega-3 fatty acids: their role in the determination of nutritional requirements and chronic disease risk. Exp Biol Med.

[65] Nuernberg K, Fischer A, Nuernberg G, Ender K, Dannenberger D (2008). Meat quality and fatty acid composition of lipids in muscle and fatty tissue of Skudde lambs fed grass versus concentrate. Small Rumin Res.

[66] Demirel G, Ozpinar H, Nazli B, Keser O (2006). Fatty acids of lamb meat from two breeds fed different forage: concentrate ratio. Meat Sci.

